# MiBio: A dataset for OCR post-processing evaluation

**DOI:** 10.1016/j.dib.2018.08.099

**Published:** 2018-09-15

**Authors:** Jie Mei, Aminul Islam, Abidalrahman Moh’d, Yajing Wu, Evangelos E. Milios

**Affiliations:** a Faculty of Computer Science, Dalhousie University, Canada; b School of Computing and Informatics, University of Louisiana at Lafayette, Lafayette, LA 70503, USA

## Abstract

We introduce a dataset for OCR post-processing model evaluation. This dataset contains fully aligned OCR texts and the ground truth recognition texts of a English biodiversity book. To better used for benchmark evaluation, we extracted the following information in TSV files: 1) 2907 OCR-generated errors with position in the OCR texts and correction in the ground truth text, 2) ground truth word and sentence segmentation of the OCR texts. In this article, we detail the data preprocessing and provide quantitative data analysis.

**Specifications table**

**Table:** 

Subject area	*Computer Science*
More specific subject area	*Natural Language Processing*
Type of data	*Text, Table*
How data was acquired	*OCR texts are generated from scanned book images by an open source OCR engine (Tesseract 3.0.2) and ground truth texts are generated with additional manual correction.*
	*Tables contain information (i.e. ground truth OCR tokens and OCR error corrections) extracted from the texts.*
Data format	*OCR & ground truth text: raw text, page separated, tokenized;*
	*Table: CSV, analyzed.*
Experimental factors	*Word/Character Error Rate of OCR Text, Levenshtein edit distance distribution of OCR errors.*
Experimental features	*OCR texts have relatively low error rate but contains complex OCR errors.*
Data source location	
Data accessibility	*Data is with this article and also in GitHub*https://github.com/jmei91/MiBio-OCR-dataset
Related research article	*Jie Mei,* Aminul Islam, Abidalrahman Moh’d, Yajing Wu, Evangelos E. Milios. *Statistical Learning for OCR Error Correction. 2017. Information Processing and Management. **in press**.*

**Value of the data**

• This dataset can be used for evaluating and comparing the performance of OCR post-processing models.
• This dataset contains page separated OCR texts and corrsponding ground truth texts. The line and paragraph breaks in the source image are preserved in both text versions. Thus each line in both OCR and ground truth texts are fully aligned and can easily refers to each other.
• OCR errors are extracted and listed in table. For each OCR error, we record its correction in the ground truth text and position in the OCR text.
• We provide the ground truth word and sentence segmentation for OCR texts to disambiguate word and sentence boundary and to be served as a reference when evaluating the tokenization performance of post-processing models.

## 1 Data

We made available Mining Biodiversity (MiBio) dataset with 2910 OCR-generated errors along with the OCR and the ground truth recognition texts for benchmark testing. The OCR text was generated from the book titled “Birds of Great Britain and Ireland (Volume II)” [1] and made publicly available by the Biodiversity Heritage Library (BHL) for Europe^1^ using Tesseract 3.0.23^2^. The ground truth text is based on an improved OCR output^3^ and adjusted manually to match with the original content of the whole book.

The scanned image data of the book contains 460 page-separated files, where the main content is included in 211 pages. The scanned images and different format of raw OCR outputs are online accessible and downloadable on https://archive.org/download/birdsofgreatbrit02butl.

## 2 Experimental design, materials, and methods

### 2.1 OCR and ground truth recognition texts preprocessing

The dataset is generated from two OCR outputs for book “Birds of Great Britain and Ireland (Volume II)” [1]. One version is generated from the standard BHL-Europe recognition workflow, which OCR technique is based on Tesseract 3.0.23. We manually correct the OCR errors in the OCR outputs to be the ground truth. We then remove footnotes and page numbers in both versions to keep the content fluency over pages.

### 2.2 OCR error extraction

When generating the error list, we adopted the following rules in extracting the OCR errors in aligned contents from the OCR and the ground truth texts:

• when segmenting an OCR-generated string into substrings that match with tokens in the ground truth text, the separating positions are approximated manually to make the best guess. For example, given an OCR string “fFrin^HluurJ” aligns with “(Fringillinæ)” in the ground truth, we separated this string into three error-correction mappings: < f →) >, < Frin^HluurJ →Fringillinæ >, and < J →) >. In another example, given an OCR string “countr}^” and “country,” in the ground truth, we split it as two error-correction mapping: < countr}^ → country > and < →, >.
• *Two ASCII substitution of unicode characters are allowed*: (æ, ae) and (Æ, AE). Note that the dataset is generated from a biodiversity book, which contains terminologies with non-English characters, for example, Corvidæ or ORIOLIDÆ. We accept these two ASCII substitutions in order to match the original terminologies to their English counterparts.
• *The aligned two words with different cases is not treated as an error.* Observed that the standard BHL-Europe recognition workflow is tend to lowercase the non-heading characters in some entirely capitalized words. Thus, we do not categorized this type of mismatchings as error. Such change in capitalization form is also hard to detect by human readers with only input text when page layout is eliminated.
• *The extra whitespaces between tokens are allowed.* It is also observed that the standard BHL-Europe recognition workflow generate extra whitespaces between tokens. We do not categorize this type of mismatch as error unless the inserted whitespace leads to a splitting or merging error.

### 2.3 OCR text tokenization

Tokenizing OCR text is one internal step in OCR post-processing. The tokenization performance affect downstream error detection and correction. Since intra-word characters of OCR errors can be misrecognized as punctuation, it is hard to disambiguate the misrecognized punctuation with true punctuation in an OCR text and thus lead to high token boundary ambiguities. We thus provide the ground truth OCR tokens for evaluating the tokenization performance of OCR post-processing models.The ground truth tokens are generated by first tokenizing the ground truth recognition text and maps the segmentation positions to the OCR texts.

Referencing to the ground truth OCR tokens in the dataset, we quantitative analysis the tokenization performance on the OCR texts by tokenization different schemes including the Whitespace, Penn Treebank, WASTE [2] and Elephant [4]. The results are shown in Table 2. The tokenization result shows that the correct word boundaries of OCR errors are hard to be identified by man-crafted rules or trained segmentation models.

**Table 2:** The performance of different tokenization schemes on the OCR text.

**Tokenization method**	**Measure [%]**			
	**Prec.**	**Rec.**	**F1**	**Err.**
**Whitespace convention**	85.5	73.14	78.84	94.93
**Penn Treebank convention**	94.33	93.94	94.13	11.08
**WASTE** (Jurish 2013)	95.18	93.14	94.15	11.05
Elephant (Evang et. al. 2013)	95.17	93.18	94.16	11.03

### 2.4 Dataset analysis

**Fig. 1:**
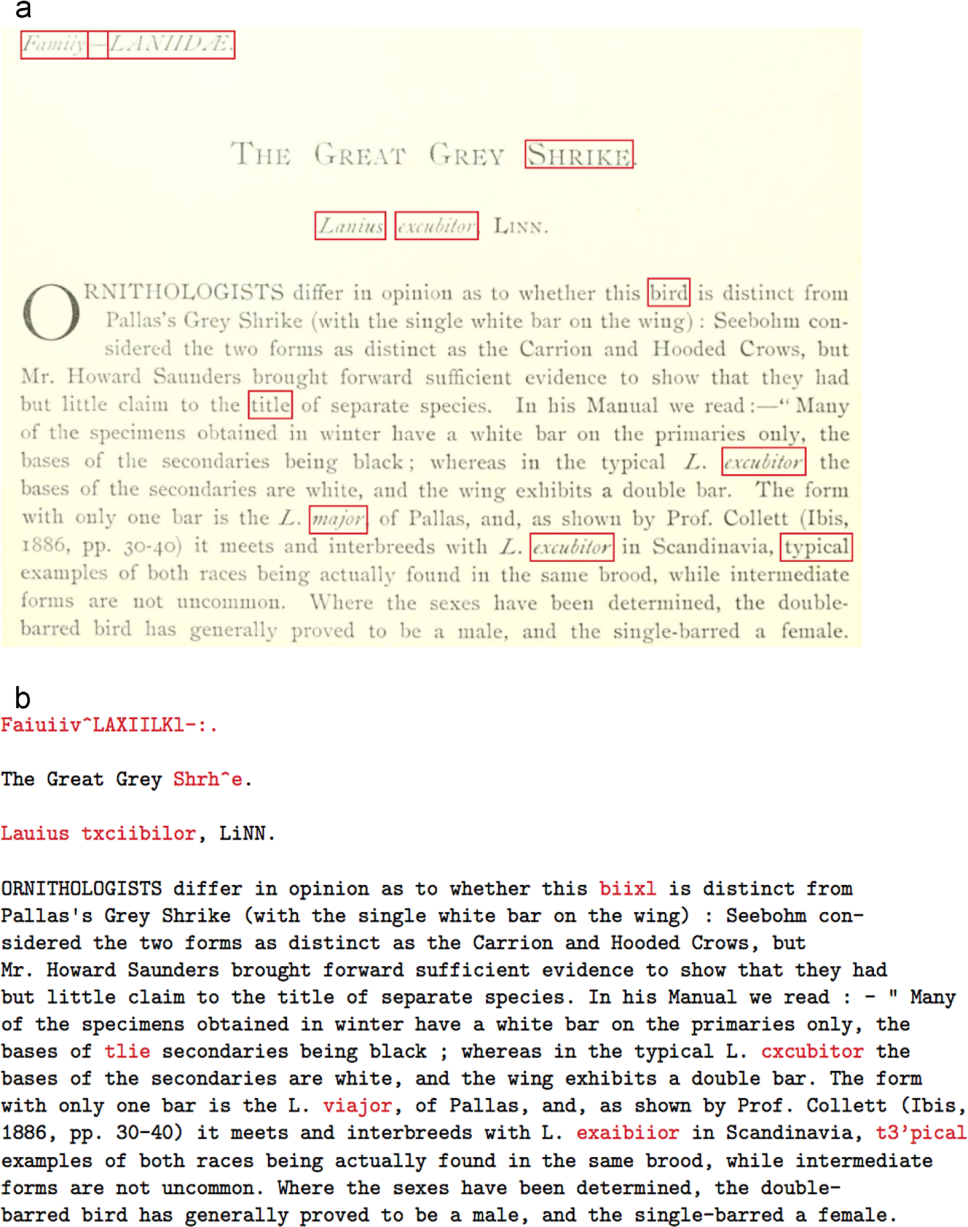
A image segment (a) and its corresponding OCR-generated text (b) of the evaluation dataset. The recognition errors are highlighted in red.

To have a close look at the OCR input/output, we sample a segment of OCR-generated text with original scanned image In Fig. 1. Table 1 shows the OCR performance, measured by precision and recall, indicating a high quality OCR output with low error rate in both word- and character-level measurements.

**Table 1:** The precision and recall of OCR generated text.

**Measure**	**Character-wise**	**Word-wise**
**Precision**	1 – 6362/409236 = 98.45%	1 – 2906/101700 = 97.14%
**Recall**	1 – 6362/407194 = 98.44%	1 – 2906/98097 = 97.04%

Observed that some OCR errors are orthographically far from their correction, we further analyze the distribution of error words with respect to Levenshtein edit distance [3] in Table 3. Although within edit distance three induces more than 80% of the OCR errors, some OCR errors have high edit distance and are very complicated to correct.

**Table 3:** The Levenshtein distance distribution of the errors in the OCR texts.

**Edit distance**	**Error statistics**		**Sample OCR error**	
	**Number**	**Percent [%]**	**Correction**	**Error**
**1**	889	30.58	galbula	ga/bula
**2**	1376	47.35	yellowish	j^ellowish
**3**	307	10.56	bents	Ijcnts
**4**	148	5.09	my	ni}’
**5**	70	2.41	Lanius	Lioiiits
**6**	51	1.75	minor	)iii>iof
**7**	28	0.96	garrulus	f;ay>///us
**8**	16	0.55	curvirostra	iUi׳7׳iyosira
**9**	5	0.17	Nucifraga	Aiiii/rut^d
**>= 10**	16	0.55	pomeranus	poiiui-iVtiis
**Total**	2906	100		

## Footnotes

1 http://www.biodiversitylibrary.org/item/35947#page/13/mode/1up

2 https://github.com/tesseract-ocr/tesseract

3 http://www.bhle.eu/en/results-of-the-collaboration-of-bhl-europe-and-impact
