# Supplementary material for: MiBio: A dataset for OCR post-processing evaluation
Source: Data Brief. 2018 Sep 15;21:251–5. doi: 10.1016/j.dib.2018.08.099 (PMC6197712; doi:10.1016/j.dib.2018.08.099)
Supplement: Supplementary file 1 — Supplementary material [file mmc1.docx]

**Conflict of interest**

All the authors confirm as no conflict of Interest.
